# How transformational leadership reduces teachers’ role stress: dual mediation of affective commitment and job satisfaction

**DOI:** 10.3389/fpsyg.2025.1634303

**Published:** 2025-08-07

**Authors:** Ping Yong, Junhui Zhang

**Affiliations:** ^1^College of International Education, Sichuan International Studies University, Chongqing, China; ^2^Department of Foreign Languages, Army Medical University, Chongqing, China

**Keywords:** transformational leadership, role stress, affective commitment, job satisfaction, primary and secondary school teachers

## Abstract

**Introduction:**

Teacher stress is a growing concern in educational settings worldwide. Grounded in the Conservation of Resources (COR) theory, this study investigates how principals’ transformational leadership influences role stress among primary and secondary school teachers. It particularly focuses on the mediating roles of affective commitment and job satisfaction.

**Methods:**

A quantitative survey was conducted among 433 teachers in China. Data were analyzed using structural equation modeling (SEM) and the PROCESS macro to explore both direct and indirect effects of transformational leadership on teacher role stress.

**Results:**

Transformational leadership was found to significantly reduce teachers’ role stress (direct effect = −0.185, 95% *CI* = [−0.277, −0.093]). Additionally, both affective commitment and job satisfaction served as significant mediators. Affective commitment exerted the strongest individual mediating effect (standardized indirect effect = −0.045, 95% *CI* = [−0.072, −0.021]), while the sequential mediation pathway involving both variables also reached statistical significance (standardized indirect effect = −0.018, 95% *CI* = [−0.031, −0.008]).

**Discussion:**

The study found that transformational leadership reduces teachers’ role stress both directly and indirectly through affective commitment and job satisfaction. These results extend the application of COR theory by identifying emotional and motivational factors as key mediators. The findings also offer practical implications for enhancing teacher well-being through school leadership development.

## 1 Introduction

The focus of school management research worldwide has gradually shifted from system efficiency to teachers’ well-being and psychological states (Collie et al., 2012, 2016; Collie, 2023; Dreer, 2023). In China, teachers in primary and secondary schools face increasingly complex professional responsibilities—ranging from instructional delivery to administrative tasks and student management—which contribute significantly to role stress (Yong and Peng, 2025). Role stress typically arises from role conflict, role ambiguity, and role overload (Rizzo et al., 1970; Nordenmark, 2004), and is considered a critical factor affecting teachers’ well-being and professional performance (Kyriacou, 2001; Papastylianou et al., 2009; Collie et al., 2016). Existing studies indicate that primary and secondary school teachers generally experience high levels of work-related stress, with notable differences based on region, educational background, teaching level, and years of experience (Liu and Onwuegbuzie, 2012; Zhang et al., 2023; Collie, 2023). Despite widespread recognition of this problem, empirical research has paid insufficient attention to how school leadership may alleviate role stress through specific psychological mechanisms.

Transformational leadership (Bass, 1985; Bass and Riggio, 2006) has consistently been associated with positive organizational outcomes, including enhanced job satisfaction, teacher engagement, and organizational commitment, particularly within educational contexts (Nguni et al., 2006; Braun et al., 2013; Yu and Jang, 2024; El Achi et al., 2025). Most of these studies have focused on its motivational functions or performance-enhancing effects. While this growing body of research highlights the constructive influence of transformational leadership on desirable outcomes, it tends to marginalize its potential role in alleviating negative psychological states such as stress or burnout. Although a few investigations have linked transformational leadership to the reduction of negative outcomes such as burnout and deviant behaviors (Khan et al., 2020; Qi et al., 2022), recent studies within educational settings suggest similar effects—for example, Tsang et al. (2022) found that transformational leadership reduced teachers’ burnout by enhancing their psychological empowerment—yet empirical evidence on its capacity to alleviate teachers’ specific role stress remains limited. This gap is especially salient in high-pressure environments such as China’s primary and secondary school systems, where teachers frequently face excessive workloads, role conflict, and role ambiguity (Liu and Onwuegbuzie, 2012; Zhang et al., 2023). Accordingly, investigating the potential of transformational leadership to buffer role-related stress holds both theoretical and practical value for the advancement of educational leadership research.

Although initial empirical evidence in China has begun to examine related psychological mechanisms—such as psychological empowerment (Tsang et al., 2022) and inclusive role identity (Wang et al., 2024)—these studies do not address teacher role stress. Moreover, a systematic review of transformational leadership in Chinese K–12 settings from 2010 to 2019 concluded that few studies have explored its psychological or affective mediating mechanisms, and constructs like role stress remain notably underrepresented in this body of research (Li, 2022).

Accordingly, this study examines how principals’ transformational leadership reduces teachers’ role stress through the dual mediation of affective commitment and job satisfaction. By integrating leadership theory with Conservation of Resources (COR) and emphasizing a mechanism-focused model, the study contributes a nuanced explanation of how school leadership can enhance teacher well-being in demanding educational contexts.

## 2 Theoretical framework and research hypotheses

### 2.1 Conservation of resources theory

This study adopts COR theory (Hobfoll, 1989, 2001, 2011) as its guiding framework. COR theory posits that individuals strive to acquire, protect, and maintain valued resources, and psychological stress occurs when these resources are threatened or lost. In educational and organizational contexts, COR theory has been widely applied to explain how leadership functions as a contextual resource that buffers against job demands (Bakker and Demerouti, 2017; Hobfoll et al., 2018).

In line with this framework, transformational leadership functions as a contextual resource that helps teachers cope with role-related stressors such as ambiguity and overload. According to COR theory, such leadership provides emotional support, goal clarity, and recognition, helping individuals conserve and build psychological resources (Hobfoll, 2011; Halbesleben et al., 2014). Empirical evidence in Chinese contexts supports this function. Tsang et al. (2022) found that transformational leadership alleviated teacher burnout by enhancing psychological empowerment among 339 primary and secondary educators. Additionally, Zhan et al. (2023) demonstrated that instructional leadership in Chinese schools indirectly reduced role stress via affective commitment, confirming that leadership acts as a resource buffer.

Under COR theory, both affective commitment and job satisfaction serve as personal psychological resources that help individuals cope with role stress (Hobfoll, 2001; Halbesleben et al., 2014). Empirically, Zhan et al. (2023) found that in Chinese primary and secondary schools, leadership reduced role stress through enhancing affective commitment. Likewise, studies in educational settings have consistently demonstrated that transformational leadership positively predicts teachers’ job satisfaction, which in turn reduces perceived work stress (Kaya, 2024). These findings affirm that both constructs are valid resource-based mediators justifying their inclusion in this study’s framework. Together, these theoretical and empirical foundations justify the proposed mediation model under the COR framework, offering a coherent explanation of how leadership builds internal resources to alleviate stress.

### 2.2 The influence of transformational leadership on teachers’ role stress, affective commitment, and job satisfaction

The theory of transformational leadership was initially introduced by Burns (1978) and later expanded by Bass (1985), emphasizing that leaders inspire individuals to exceed expectations by shaping a vision, stimulating higher-order needs, and fostering organizational trust. Geijsel et al. (2003) highlighted the critical role of transformational leadership in complex educational reforms, as it enhances teachers’ commitment and capabilities, thereby facilitating organizational change in schools. More recently, Berkovich (2016) emphasized the need to contextualize transformational leadership theory within educational environments, noting both its influence on teacher engagement and its limitations in dynamic school settings.

Empirical studies suggest that transformational leadership can improve school climate, enhance teachers’ professional identity, and reduce work-related conflicts, which in turn alleviates teachers’ role stress (Alzoraiki et al., 2024; Kovjanic et al., 2013; Lin et al., 2022; Tsang et al., 2022). From the COR perspective, From the COR perspective, transformational leadership provides key contextual resources—including vision clarity, emotional support, and individualized recognition—that help teachers better manage their role expectations and reduce resource loss (Halbesleben et al., 2014; Hobfoll, 1989).

Accordingly, we hypothesize:H1: Principals’ transformational leadership negatively influences teachers’ role stress.

Transformational leadership also plays an important role in cultivating internal psychological resources. Affective commitment reflects emotional attachment and identification with the school, which fosters greater investment in work roles and resilience in stressful contexts (Meyer and Allen, 1991; Klassen and Chiu, 2011; Kılınç et al., 2024). Studies in educational settings have consistently shown that transformational leadership fosters affective commitment by conveying shared purpose and individual care (Keskes et al., 2018; Nguni et al., 2006; Ross and Gray, 2006; Shao et al., 2022).

H2: Principals’ transformational leadership positively influences teachers’ affective commitment.

Job satisfaction is another internal resource that enhances positive affect and buffers stress. Teachers with high job satisfaction experience fewer negative outcomes such as burnout and role conflict (Bogler and Somech, 2004; Kaya, 2024; Lei et al., 2024). Transformational leadership improves satisfaction by empowering teachers and enhancing role clarity (Aydın et al., 2013; Polatcan and Cansoy, 2019; Bibi et al., 2022).

H3: Principals’ transformational leadership positively influences teachers’ job satisfaction.

### 2.3 The mediating role of affective commitment and job satisfaction

Affective commitment has been shown to reduce emotional exhaustion and role overload by increasing teachers’ engagement and willingness to navigate job demands (Kaur, 2020; Washburn et al., 2022). Teachers with stronger affective commitment are more likely to reframe challenges positively and persevere in difficult situations, thereby reducing role stress.

H4: Affective commitment partially mediates the relationship between principals’ transformational leadership and teachers’ role stress.

Job satisfaction functions similarly by promoting positive appraisal and work engagement, which leads to lower perceived role conflict and ambiguity (Collie et al., 2012; Jaafar, 2021; Singh et al., 2019).

H5: Job satisfaction partially mediates the relationship between principals’ transformational leadership and teachers’ role stress.

From a sequential mediation perspective, affective commitment and job satisfaction may jointly transmit the influence of transformational leadership on role stress. According to COR theory, transformational leadership first strengthens teachers’ emotional attachment (i.e., affective commitment), which in turn enhances their positive affect and intrinsic motivation, leading to increased job satisfaction (Meyer and Allen, 1991; Klassen and Chiu, 2011). Higher job satisfaction enables teachers to appraise work demands more positively, thereby reducing role stress (Collie et al., 2012; Choi and Kang, 2021). This sequential pathway highlights how psychological and affective resources are built progressively, buffering stress in a layered manner (Hobfoll, 2001; Halbesleben et al., 2014). Therefore, we propose the following hypothesis:

H6: Affective commitment and job satisfaction sequentially mediate the relationship between principals’ transformational leadership and teachers’ role stress.

### 2.4 Research framework

**FIGURE 1:**
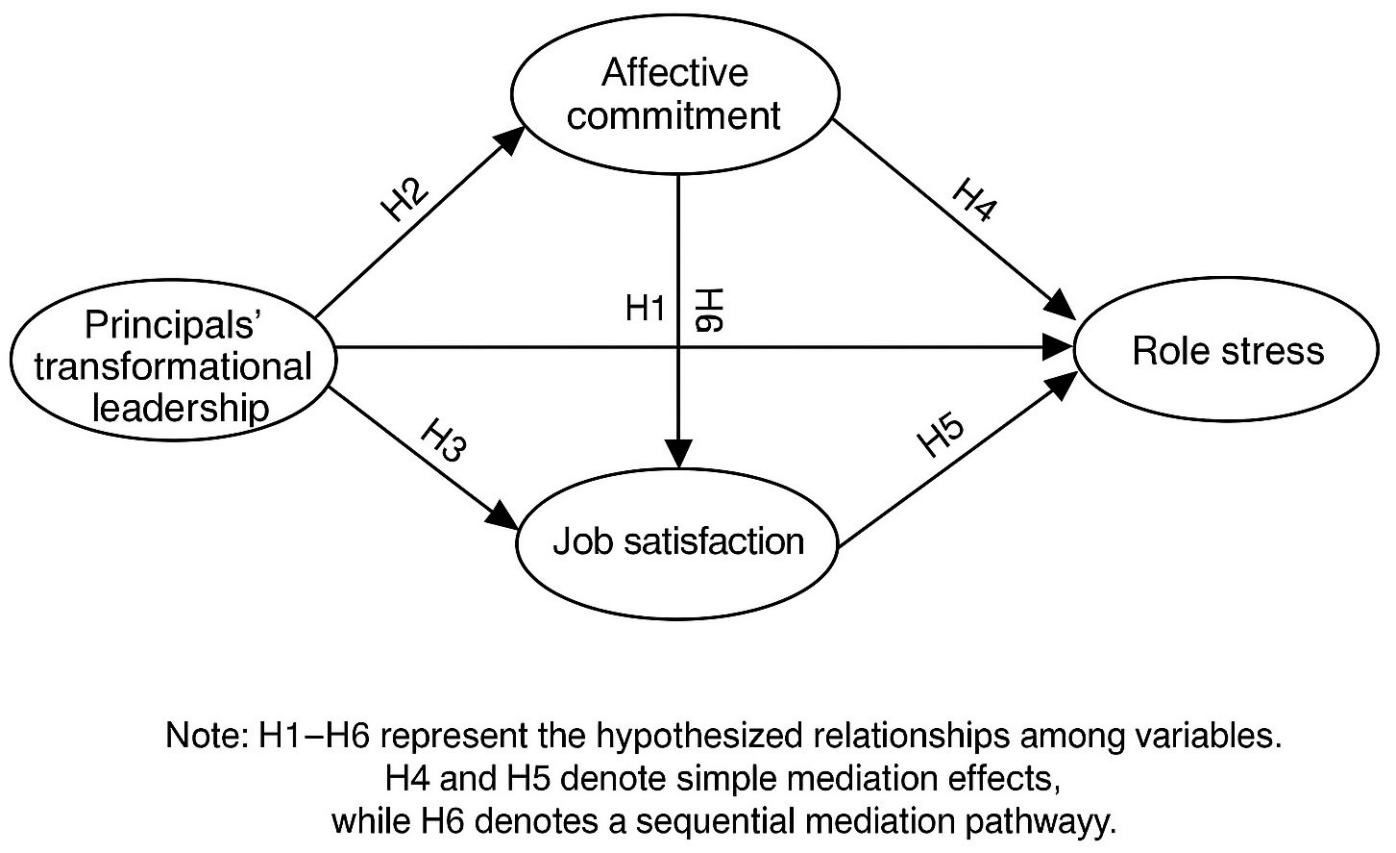
Research model of the direct and indirect effects of transformational leadership on role stress.

As illustrated in Figure 1, the model proposes that transformational leadership reduces teachers’ role stress through the dual mediation of affective commitment and job satisfaction.

## 3 Research methodology

### 3.1 Data collection and sample

This study aims to examine the direct and indirect effects of principals’ transformational leadership on teachers’ role stress, with a particular focus on the mediating roles of affective commitment and job satisfaction. A cross-sectional survey design was employed, and data were collected using a structured questionnaire to test the proposed hypotheses. Additionally, the Bootstrap method with 5,000 resamples was used to test the mediating effects of affective commitment and job satisfaction in the relationship between transformational leadership and role stress, ensuring the robustness of the estimation results.

The study targeted primary and secondary school teachers in City C, China. This location was selected due to its rapid educational development and recent policy-driven reforms, which have significantly increased the workload and responsibilities of local teachers. City C features a diverse mix of urban and suburban schools, including both public and private institutions, making it a representative context for investigating how leadership practices impact teacher well-being. The city’s varied teacher demographics and wide range of school types also enhance the external validity and generalizability of the findings.

To improve representativeness, we employed a stratified random sampling method. The sample was stratified based on key demographic and organizational variables, including school type (primary vs. secondary), school location (urban vs. suburban), and teacher characteristics such as gender, years of teaching experience, and educational background. Within each stratum, participants were randomly selected to ensure proportional representation across different subgroups. A total of 502 questionnaires were distributed, with 433 valid responses collected, resulting in an effective response rate of 86.2%. Among the valid respondents: Gender distribution: 360 female teachers (83.1%) and 73 male teachers (16.9%). Educational background: 45 teachers (10.4%) had a bachelor’s degree or lower, 308 teachers (71.1%) held a bachelor’s degree, and 80 teachers (18.5%) had a master’s degree or higher. School type: 263 respondents (60.7%) were primary school teachers, while 170 (39.3%) were secondary school teachers.

This study adhered to strict academic ethical guidelines. Before participation, all respondents were fully informed about the research objectives, and participation was entirely voluntary. The study ensured participants’ privacy and data confidentiality, with all collected data used solely for research purposes. Additionally, the questionnaire survey received approval from the institutional ethics committee, ensuring compliance with ethical research standards.

### 3.2 Variable measurement

This study employed a structured questionnaire for data collection, consisting of five sections:

Demographic Characteristics: Participants provided information on gender, age, education level, teaching experience, and school type.

Transformational Leadership Scale: The Chinese Transformational School Leadership Questionnaire developed by Liu (2013) was used, which consists of four dimensions and 29 items, measured on a 5-point Likert scale ranging from 1 (strongly disagree) to 5 (strongly agree). This scale has been widely used and validated in Chinese educational settings. In this study, the Cronbach’s alpha was 0.96.

Affective Commitment Scale: The affective commitment subscale from the Organizational Commitment Questionnaire developed by Meyer et al. (1993) was adopted, consisting of six items, measured on a 5-point Likert scale. This scale has been translated into Chinese and applied in previous studies involving Chinese teachers. The Cronbach’s alpha for this study was 0.90.

Job Satisfaction Scale: The job satisfaction scale translated by Shu and Liang (2015) from Hackman and Oldham (1974) was used, comprising three items, measured on a 5-point Likert scale. This translated scale has demonstrated good reliability in prior Chinese samples. In this study, the Cronbach’s alpha was 0.82.

Role Stress Scale: Role stress was assessed using a 13-item scale originally developed by Peterson et al. (1995), and revised for Chinese educational settings by Li and Zhang (2005). This scale measures role conflict, role ambiguity, and role overload, consisting of 13 items, measured on a 5-point Likert scale. The Cronbach’s alpha in this study was 0.95.

## 4 Research results

### 4.1 Common method bias (CMB) test

To minimize the impact of CMB, this study implemented several control measures during both the data collection and analysis phases. During data collection, anonymous questionnaires were used, along with reverse-coded items and randomized question order to reduce respondents’ subjective biases. For data analysis, Harman’s single-factor test was conducted to detect CMB. The results showed that the largest single factor accounted for 32.5% of the total variance, which is below the 40% threshold commonly recommended to indicate the presence of substantial common method variance (Podsakoff et al., 2003). These results suggest that CMB is unlikely to pose a serious threat to the validity of the study’s findings.

### 4.2 Exploratory factor analysis (EFA)

To evaluate the construct validity of the measurement instrument, an EFA was conducted using principal component analysis with Promax rotation. The Kaiser-Meyer-Olkin (KMO) measure of sampling adequacy was 0.955, exceeding the recommended threshold of 0.80, indicating that the data were suitable for factor analysis. Bartlett’s Test of Sphericity was also significant (χ^2^ = 17964.641, df = 1275, *p* < 0.001), supporting the factorability of the correlation matrix.

Using the eigenvalue-greater-than-one criterion and scree plot analysis, nine common factors were extracted, explaining a total of 85.556% of the cumulative variance. All items demonstrated high factor loadings (≥0.737), exceeding the recommended threshold of 0.40, with no cross-loadings observed. This confirmed the convergence and discriminant validity of the measurement items across factors.

Items clustered consistently with their theoretical constructs: transformational leadership (items XA1–XD5), affective commitment (M11–M16), job satisfaction (M21–M23), and role stress (YA1–YC5), supporting the intended multidimensional structure of the scale. No item deletion was required, and all items retained strong psychometric properties. A summary of representative items and factor loadings is presented in Table 1.

**TABLE 1:** Factor loadings from exploratory factor analysis (EFA).

*Item*	*Factor loading*	*Factor name*
*XA2*	*0.86*	*Transformational leadership*
*XB5*	*0.87*	
*XC1*	*0.88*	
*XD4*	*0.92*	
*M11*	*0.85*	*Affective commitment*
*M21*	*0.84*	*Job satisfaction*
*YA1*	*0.83*	*Role stress*
*YB1*	*0.88*	
*YC1*	*0.91*	

Factor loadings ≥0.40 are displayed.

### 4.3 Descriptive statistical analysis

The means, standard deviations, and correlation coefficients for all variables are presented in Table 2. The results indicate that principals’ transformational leadership is positively correlated with teachers’ affective commitment and job satisfaction (*p* < 0.01) while showing a significant negative correlation with teachers’ role stress (*p* < 0.01). Additionally, affective commitment is positively correlated with job satisfaction but negatively correlated with role stress. Similarly, job satisfaction is significantly negatively correlated with role stress. These preliminary findings provide initial support for the research hypotheses.

**TABLE 2:** Means, standard deviations, and correlation coefficients of variables.

Variable	*M*	*SD*	1	2	3	4
1. Transformational leadership (TL)	3.65	0.80	1			
2. Affective commitment (AC)	3.80	0.84	0.21**	1		
3. Job satisfaction (JST)	3.54	0.94	0.19**	0.31**	1	
4. Role stress (RS)	3.64	0.86	−0.27**	−0.34**	−0.39**	1

TL, Transformational Leadership; AC, Affective commitment; JST, Job satisfaction; RS, Role stress. **p < 0.01.

### 4.4 Reliability and validity analysis

**FIGURE 2:**
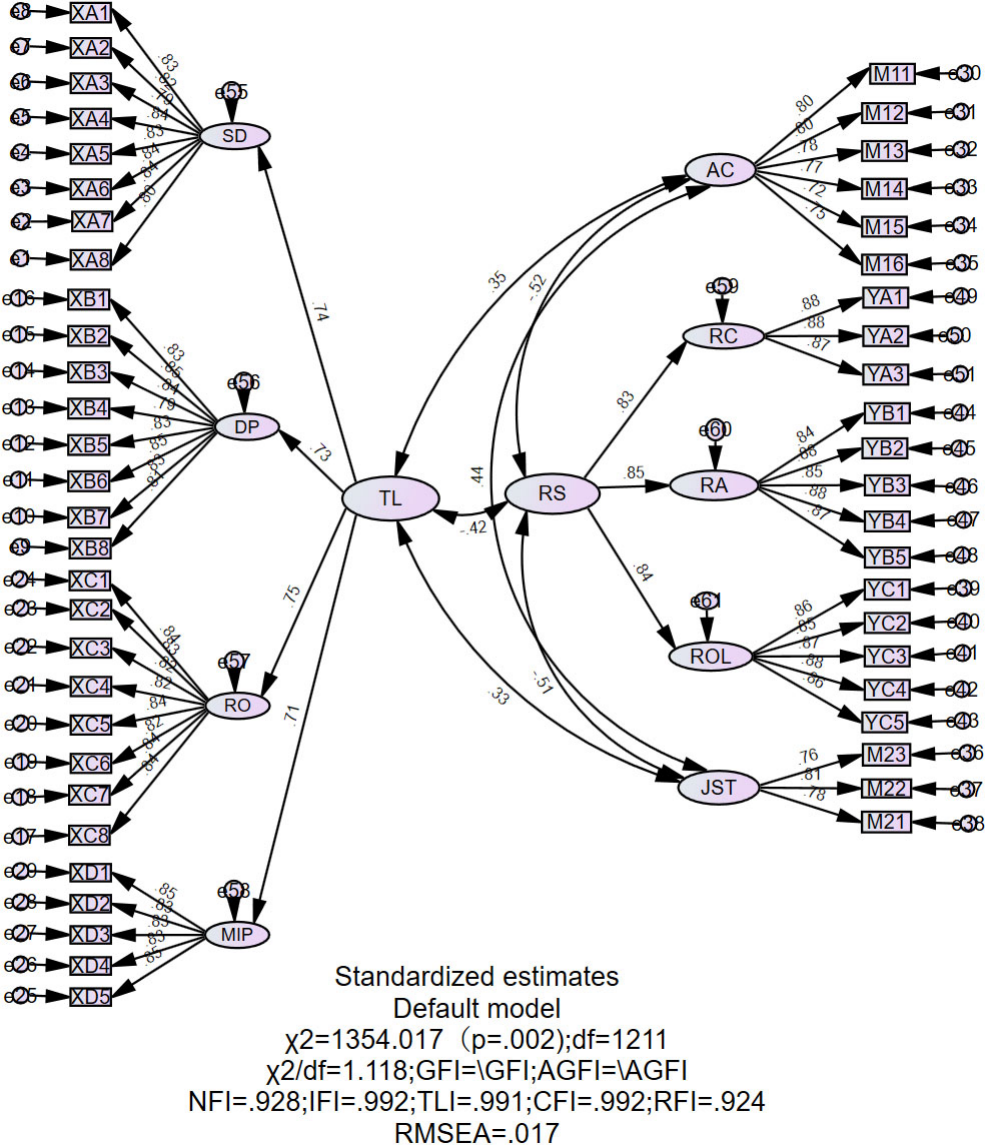
The CFA model for measurement validity.

We conducted structural equation modeling (SEM) using AMOS 26.0 to test the hypothesized model fit and evaluate the measurement model’s reliability and validity. The model fit was assessed using multiple indices, including the Normed Fit Index (NFI), Incremental Fit Index (IFI), Relative Fit Index (RFI), Comparative Fit Index (CFI), Tucker–Lewis Index (TLI), Root Mean Square Error of Approximation (RMSEA), and Standardized Root Mean Square Residual (SRMR). The results indicate that the model fit indices demonstrate a good fit (*CMIN/DF* = 1.118, *RMSEA* = 0.017, *NFI* = 0.903, *IFI* = 0.992, *TLI* = 0.991, *CFI* = 0.992, *RFI* = 0.924), supporting the adequacy of the measurement model. The CFA results are presented in Figure 2.

The reliability and validity of the measurement model were assessed using Composite Reliability (CR) and Average Variance Extracted (AVE). As shown in Table 3, all latent variables had CR values exceeding 0.70 and AVE values greater than 0.50, meeting the criteria proposed by Fornell and Larcker (1981). These results indicate that the observed variables adequately explain their respective latent constructs, demonstrating strong convergent validity.

**TABLE 3:** Reliability and validity analysis of measurement instruments.

Variable	*CR*	*AVE*	*MSV*	*MaxR (H)*	TL	AC	JST	RS
TL	0.82	0.54	0.17	0.83	0.74			
AC	0.90	0.59	0.27	0.90	0.35***	0.77		
JST	0.83	0.61	0.26	0.83	0.33***	0.44***	0.78	
RS	0.88	0.71	0.27	0.88	−0.42***	−0.52***	−0.51***	0.84

CR, Composite Reliability; MaxR (H), Maximum Reliability (H). ***p < 0.001.

Discriminant validity was evaluated following Fornell and Larcker’s (1981) criteria. The results showed that the square root of each AVE exceeded its correlations with other constructs, and the Maximum Shared Variance (MSV) was lower than the AVE, confirming that all latent variables exhibit sufficient discriminant validity.

Overall, these findings suggest that the measurement model has high reliability and validity, providing a solid foundation for further data analysis in this study.

### 4.5 SEM analysis

**FIGURE 3:**
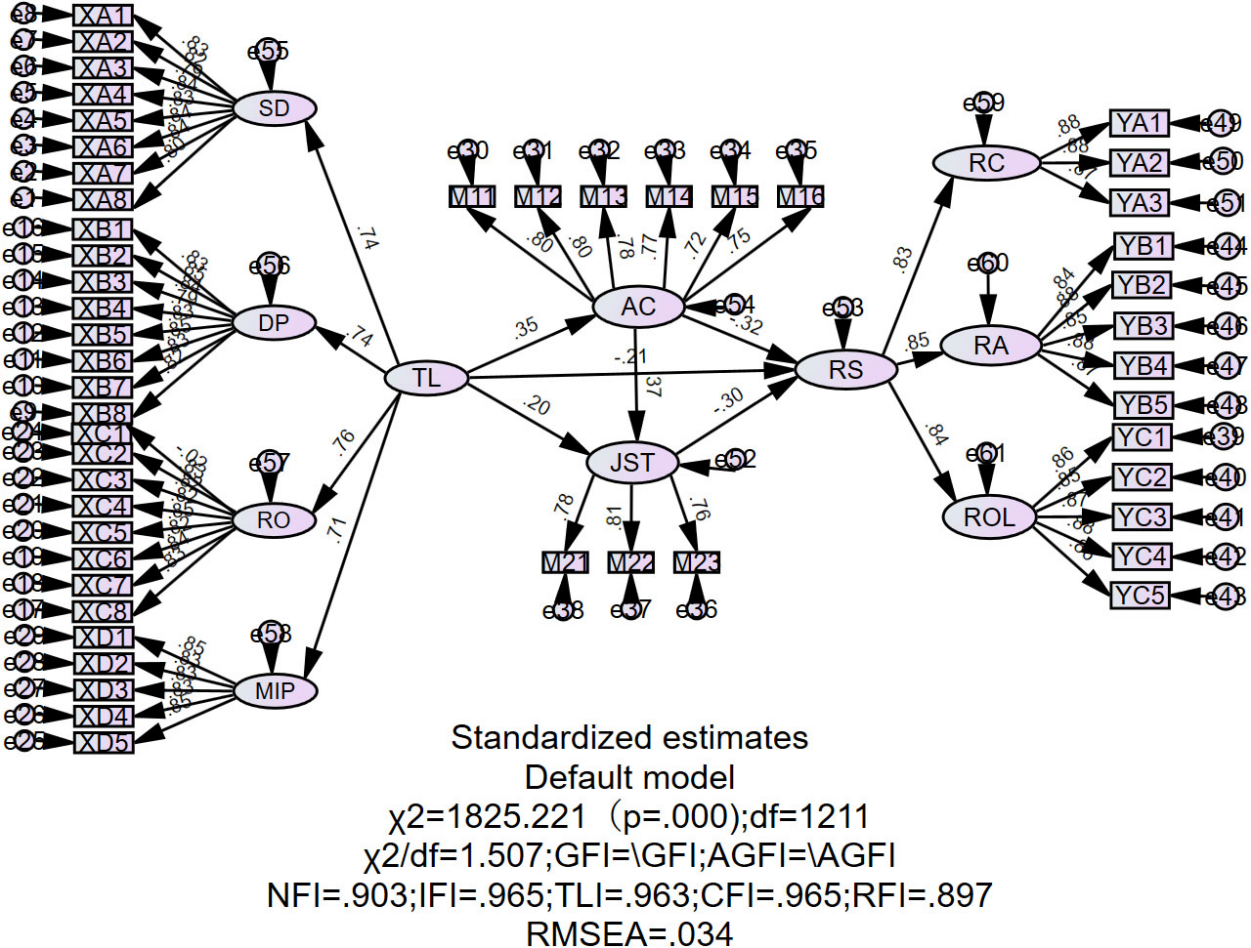
Structural equation model of the effects of transformational leadership on teachers’ role stress.

This study employed the maximum likelihood estimation (MLE) method to test the proposed structural equation model using AMOS 26.0. As shown in Figure 3, the model fit indices demonstrated good fit: χ^2^ = 1825.221, *df* = 1211, χ^2^/df = 1.507, *p* < 0.001, *CFI* = 0.965, *RMSEA* = 0.034, and *SRMR* = 0.080. These values meet the commonly recommended thresholds (Hu and Bentler, 1999), supporting the adequacy of the model.

The model fit indices indicate a good overall model fit, supporting the validity of the proposed model, as shown in Table 4.

**TABLE 4:** Structural model fit indices.

Model	χ^2^	*df*	χ^2^/*df*	*p*-*value*	*CFI*	*RMSEA*	*SRMR*
	1825.22	1211	1.51	<0.001	0.97	0.03	0.08

χ^2^, chi-square; df, degrees of freedom; CFI, Comparative Fit Index; RMSEA, Root Mean Square Error of Approximation; SRMR, Standardized Root Mean Square Residual.

### 4.6 Mediation effect test

This study used PROCESS Model 6 (Hayes, 2018) to examine the serial mediation effect of affective commitment and job satisfaction between transformational leadership and teachers’ role stress. The Bootstrap method (5,000 resamples, 95% *CI*) was applied to ensure result robustness. Mediation is considered significant if the confidence interval (*Boot LLCI–Boot ULCI*) does not contain zero. Table 5 presents the results.

**TABLE 5:** Bootstrap analysis of serial mediation effects.

Effect		Bootstrapping					
		*Effect*	*SE*	*T*	*P*	*LLCI*	*ULCI*
Total effect of X on Y		−0.293	0.05	−5.88	0.000	−0.391	−0.195
Direct effect of X on Y		−0.185	0.047	−3.937	0.000	−0.277	−0.093
Indirect effect	TOTAL	−0.101	0.022			−0.145	−0.058
	Ind1 TL-AC-RS	−0.045	0.013			−0.072	−0.021
	Ind2 TL-JST-RS	−0.038	0.014			−0.066	−0.012
	Ind3 TL-AC-JST-RS	−0.018	0.006			−0.031	−0.008
Comparison of mediation paths	Ind1-Ind2	−0.007	0.019			−0.044	0.030
	Ind1-Ind3	−0.028	0.012			−0.530	−0.005
	Ind2-Ind3	−0.021	0.015			−0.050	0.008

The results indicate that transformational leadership has a significant total effect on teachers’ role stress, supporting H1. After controlling for mediating variables, the direct effect remains significant, suggesting that transformational leadership continues to directly influence role stress. Additionally, the total indirect effect is significant, indicating the presence of multiple mediation pathways, supporting H2, H3, and H4. Specifically, both single mediation paths are significant, with affective commitment playing a more prominent role. The sequential mediation effect is relatively smaller but remains significant.

In terms of standardized estimates, transformational leadership significantly increased affective commitment (β = 0.51, *p* < 0.001) and job satisfaction (β = 0.44, *p* < 0.001), while both mediators negatively predicted role stress (AC → RS: β = −0.27, *p* < 0.001; JST → RS: β = −0.23, *p* < 0.001). The sequential path TL → AC → JST → RS also yielded a small but significant effect (β = −0.018, 95% *CI* [−0.031, −0.008]). These results align with COR theory, suggesting that transformational leadership cultivates internal psychological resources that buffer against role-related stress. Affective commitment played a stronger mediating role than job satisfaction, highlighting the importance of emotional attachment in helping teachers cope with occupational stress.

Overall, the findings suggest that affective commitment and job satisfaction partially mediate the relationship between transformational leadership and role stress, with affective commitment being the stronger mediator.

## 5 Discussion

Grounded in COR theory, this study examined the impact of transformational leadership on teachers’ role stress and analyzed the mediating roles of affective commitment and job satisfaction. The findings indicate that transformational leadership effectively reduces teachers’ role stress, with affective commitment and job satisfaction partially mediating this relationship.

Based on these results, this section provides an in-depth discussion of the findings, highlights the theoretical contributions and practical implications, and proposes directions for future research.

### 5.1 Discussion of findings

This study found that transformational leadership significantly and negatively predicts teachers’ role stress. This confirms prior research suggesting that supportive and motivating leadership styles reduce stress related to role ambiguity, conflict, and overload (Leithwood and Jantzi, 2006; Tsang et al., 2022). Under the COR theory (Hobfoll, 1989, 2001), leadership acts as a contextual resource by providing goal clarity, emotional support, and recognition—key mechanisms that help conserve psychological energy and reduce resource depletion (Halbesleben et al., 2014).

Consistent with this, the study further identified affective commitment and job satisfaction as significant mediators. This aligns with existing COR-based studies, which argue that emotional attachment (i.e., affective commitment) and positive work evaluations (i.e., job satisfaction) function as personal resources that mitigate stress responses (Kaya, 2024; Zhan et al., 2023). Transformational leaders cultivate trust and a sense of purpose, which help strengthen teachers’ affective commitment and job satisfaction. These strengthened internal resources, in turn, serve to reduce perceived role stress. This pathway aligns with Meyer and Allen’s (Meyer and Allen, 1991) model, which conceptualizes affective commitment as a motivational force, and Collie et al.’s (2012) findings that job satisfaction enhances teacher well-being.

However, while the findings align with COR theory, minor contextual differences emerge. For instance, the mediating strength of affective commitment may be heightened in collectivist cultures like China’s, where emotional bonds to institutions are valued more highly than in Western contexts (Hofstede, 2001). Such cultural factors may explain stronger indirect effects observed in this study compared to Western-based research.

Theoretically, this study contributes by integrating COR theory with educational leadership, clarifying not just the presence of effects, but their resource-based mechanisms. It advances the literature by empirically validating that transformational leadership not only exerts direct effects, but also builds psychological resources that mediate stress pathways.

### 5.2 Theoretical contributions

This study makes several theoretical contributions:

First, it introduces a sequential mediation framework, demonstrating how transformational leadership alleviates teachers’ role stress via two successive psychological resources: affective commitment and job satisfaction. While prior studies have explored related mechanisms—for instance, Choi and Kang (2021) tested a serial mediation linking leadership to teacher self-efficacy, and Kaya (2024) examined job satisfaction and professional resilience as mediators in Chinese educational settings—few have directly modeled dual bridging paths between leadership and stress reduction. This study extends the literature by integrating both mediators within a unified explanatory framework. It also responds to calls for more nuanced, multistep models in high-demand educational contexts (Tsang et al., 2022). Furthermore, Leithwood and Sun’s (2012) meta-review emphasized the influence of transformational leadership on teacher outcomes through complex mediating structures; however, few studies have empirically validated such mechanisms in stress-alleviation contexts. By addressing this gap, the current study refines the conceptualization of transformational leadership pathways in teacher stress management.

Second, drawing on COR theory, this study clarifies how transformational leadership enhances teachers’ psychological resource reserves to effectively manage role stress. COR theory posits that individuals cope with stress by acquiring and preserving valued resources. By demonstrating the positive impact of transformational leadership on these internal resources, the study offers a concrete explanation of how leadership behavior buffers teachers against role demands. While prior research by Kaya (2024) and Tsang et al. (2022) supported resource-based interpretations, Hobfoll et al. (2018) highlighted that stress coping involves layered and sequential resource accumulation—a notion directly reflected in the dual-mediation model tested here. Thus, this study both extends and deepens the theoretical application of COR theory in educational stress contexts.

Finally, this study contributes to the growing recognition of school leadership as a proactive intervention for teacher stress. Much of the prior literature has focused on the antecedents or symptoms of stress, while relatively few have examined leadership’s role in shaping adaptive psychological resources. Liu (2018), in a comprehensive review of transformational leadership research in China, noted the limited empirical attention to mediating and moderating mechanisms, particularly at the individual psychological level. By empirically validating affective commitment and job satisfaction as internal mediators, our study addresses this gap in the Chinese context and provides localized evidence for designing culturally grounded school-level interventions that support teachers’ psychological well-being.

### 5.3 Practical implications

The findings of this study provide significant practical insights for school administrators and educational policymakers, highlighting the critical role of transformational leadership in alleviating teachers’ role stress, enhancing job satisfaction, and fostering professional well-being.

First, school administrators should actively foster transformational leadership practices by creating a shared school vision, offering individualized support, and encouraging professional innovation. This can be operationalized through structured one-on-one mentoring programs, leadership development workshops, and regular professional feedback meetings. Schools should also implement goal alignment initiatives that connect teachers’ personal development plans with the school’s strategic objectives, thereby reinforcing their sense of value and commitment.

Second, strengthening teachers’ affective commitment and job satisfaction is key to mitigating stress. Leaders should cultivate a supportive work environment by enhancing teacher autonomy, recognizing contributions, and involving teachers in decision-making. Practical strategies include establishing peer-nominated teacher award programs, expanding opportunities for teachers to serve on school governance committees, and creating transparent feedback channels that validate teachers’ voices in curriculum and policy development.

Third, policymakers should support schools in building a systemic framework for teacher stress management. This includes promoting data-informed workload monitoring platforms, refining class scheduling procedures, and instituting early-warning systems for burnout. Additionally, educational departments can mandate stress management modules in teacher training, expand access to school-based mental health services, and incentivize schools that implement innovative teacher wellness programs.

Collectively, these strategies offer a holistic and sustainable framework for schools and policymakers to proactively reduce teachers’ role stress, enhance professional well-being, and improve overall educational quality in increasingly demanding educational environments.

### 5.4 Research limitations and future directions

Despite its contributions, this study has certain limitations that suggest directions for future research.

First, this study adopted a cross-sectional design, which limits the ability to establish causal relationships. Future studies could employ a longitudinal research design to better capture the long-term effects of transformational leadership on teachers’ role stress and provide more robust evidence for causality.

Second, the sample was drawn exclusively from primary and secondary school teachers in Southwest China, meaning that the findings may be influenced by regional cultural contexts and educational policies. Future research could expand the scope to other regions and international samples to examine the generalizability of the findings across diverse educational and policy environments.

## 6 Conclusion

Grounded in COR theory, this study examined the impact mechanism of transformational leadership on teachers’ role stress and confirmed the mediating roles of affective commitment and job satisfaction. The findings suggest that transformational leadership indirectly reduces role stress by strengthening teachers’ affective commitment and enhancing job satisfaction.

These findings not only extend the application of transformational leadership theory in the educational domain but also provide practical guidance for school administrators, offering insights into leadership strategies that can effectively reduce teachers’ role stress and improve their overall well-being.

## Data Availability

The original contributions presented in this study are included in this article/supplementary material, further inquiries can be directed to the corresponding author.
